# Research on a General SER Rate Prediction Model Based on a Set of Configuration Parameters Related to SER

**DOI:** 10.3390/mi16080950

**Published:** 2025-08-19

**Authors:** Shougang Du, Shulong Wang, Shupeng Chen

**Affiliations:** 1 China Academy of Space Technology, Beijing 100086, China; 2 Wide Bandgap Semiconductor Technology Disciplines State Key Laboratory, Xidian University, Xi’an 710071, China; slwang@xidian.edu.cn (S.W.); spchen@xidian.edu.cn (S.C.)

**Keywords:** SER, RPP, IRPP, LET, MC, SER rate prediction

## Abstract

This article comprehensively analyzes the new developments and challenges faced by several typical prediction models in the field of radiation effects in recent years. The models discussed include the RPP model, the extended RPP (rectangular parallelepiped) model, and the IRPP (integral rectangular parallelepiped) model. The article conducts a comprehensive analysis of the limitations of the assumption that uses the linear energy transfer (LET) of incident particles and the SEU (single-particle upset) cross-section (without considering the energy and type of ions) to predict the rate of single-particle effects (SEUs). Additionally, the article points out that with the continuous progress of integrated circuit technology, the geometric shape of the target circuit, the energy of the incident particles, the type of particles, and more precise physical models corresponding to the interaction between radiation and matter have become increasingly important in evaluating the sensitivity to single-particle effects (SEEs). Subsequently, based on the probability characteristics of SEE, a series of general estimation equations for the SEE rate are derived, considering particle energy, particle type, and the probability of influence at a specific moment. Then, by introducing the concept of interaction volume, the concept of sensitive volume is further expanded, and using these general equations, the relationship between the SEE rate cross-section and the SEE projected area is derived, simplifying the SEU rate prediction equation to a form that can be directly used in engineering applications. Finally, the article emphasizes a complete method of applying the general prediction equation to engineering to estimate the radiation disturbance performance of two typical verification circuits, and provides the corresponding prediction results.

## 1. Introduction

Particle radiation, whether from space and Earth’s environment or from artificial sources such as particle accelerators, radioactive particle sources, and nuclear radiation environments, can affect the normal operation of electronic components. When the effects are caused by the passage of a single particle rather than the cumulative effect of many particles, it is referred to as SER [1]. SER is induced by the random incidence of particles of different types, energies, and angles of incidence [2]. Since the early recognition of SER as a factor influencing reliability in space and terrestrial environments, the observed single-event effects have been classified into two main categories: soft errors (such as Single-Event Upsets or Single-Event transients) and hard errors (such as Single-Event Latch Up, Single-Event Burnout, Single-Event Gate punch-through, etc.). Furthermore, various methods have been developed, such as the RPP and IRPP models, to measure and predict their frequency [3,4,5,6,7,8,9,10,11,12,13].

Up to now, the most widely used prediction models and computer codes for SER Rate are the RPP and IRPP models [13,14,15,16], which are based on energy deposition and charge collection. These models have been factually established for nearly 40 years to predict the error rates of different process circuits or devices in various space environments [17]. Over the years, their extensive usability and effectiveness have been validated through continuous verification with on-orbit data, demonstrating their fundamental quality and prompting ongoing improvements in their past applications [18].

The RPP model is simplified into a single integral about chord length and LET distribution by adopting many assumptions that are independent of device size and difficult to prove physically, realizing the simplicity and ease of use of the prediction model form [19]. These assumptions are the basis of the RPP prediction model. If any of them is in doubt, the prediction results will be suspicious [20,21,22,23,24,25,26]. Moreover, since the RPP model is a single sensitive model, any generalization and expansion of more complex systems will propagate its assumptions. The RPP model is based on energy deposition, because in the absence of such additional information as device structure or circuit topology, energy (or equivalent charge) is the only available effect characterization quantity. The correlation between the deposited energy and the circuit SER effect is realized by a single sensitive body. Therefore, an effective extension of the RPP model is to redefine the sensitive body.

The basic new physical basis of the IRPP model compared with the RPP model lies in the relaxation of the assumption that the inversion can be characterized by a single critical charge or effective energy [18]. The IRPP model assumes that there is a series of critical energies due to changes between cells in the measurement. In this case, the measurement cross-section of an integrated circuit composed of many nominally identical cells (such as memory) will deviate from the expected result of a single cell.

Essentially, the randomness of the radiation effect is attributed to the initial radiation and subsequent radiation transfer, and the circuit is assumed to be deterministic. Here, we first demonstrate a series of general equations (Monte Carlo simulation and engineering test evaluation) that can be used to predict the rate of SER from the perspective of particle energy, particle type, and effect probability at a certain time, and these equations can be simplified into familiar analytical forms when making sufficiently extreme simplifying assumptions. The strength of the MC (Monte Carlo) method is that even without these simplified assumptions, it is still treatable, which in principle can achieve more accurate estimates. Then, the concept of sensitive body is extended, the concept of interaction body is introduced, and the corresponding relationship between the SER rate cross-section and the SER projected area is revealed by using the general equation. The SER Rate prediction equation is simplified into an analytical form that can be directly used in engineering. Finally, the complete idea and the prediction results of the radiation resistance performance of two typical verification circuits by using the prediction equation in engineering are emphatically provided.

## 2. Mathematical Foundations of Rate Prediction

The key indicator for analyzing SER is the SER Rate in a given radiation environment. The purpose of this section is to describe a comprehensive mathematical framework that can be used for predicting the SER Rate, expanding upon the approach first proposed by Weller et al. [27].

### 2.1. General Single-Event Effect Rate Equation

Peterson et al. have conducted a thorough review of the mathematical foundations for predicting SER Rate [18]. However, the computational methods they described begin with a direct and deterministic relationship between energy deposition and the properties of the incident particles. In this work, we employ a general model to analyze the prediction of SER Rate, which allows us to calculate the probability of any effect caused by a single quantum of radiation. In this model, the term “event” is used to describe the interaction between the radiation quantum and the system being analyzed. The process initiated by a single radiation quantum is referred to as an “effect.” For each event, there exists a probability associated with this “effect”. The foundation of predicting SER Rate is based on the calculus of these probabilities.

First, suppose that the research subjects, such as sample devices, circuits, or systems, exist within or around a spatial region referred to as the “world”. Assume that the world is a convex body, and the surface normal unit vector at position $\vec{x}$ is denoted as $\hat{n}\left(\vec{x}\right)$.

The research subjects are relatively stationary in the world reference system. The size of the world is unspecified with respect to both the absolute size and the relative size of the research object, and it is assumed that the occurrence of effects is entirely determined by the physical processes occurring within the world.

According to the sources of particle radiation surrounding the research subjects, particle radiation sources can be divided into two independent categories: particles entering the world from external sources, such as cosmic rays, and particles originating from within the world itself, such as those produced by spontaneous radioactive decay.

The external radiation source is completely specified at every point on the world surface by the flux distributions $\hat{u}\Phi \left(z,E,\hat{u},\vec{x},t_{x}\right)$. The internal radiation source is specified by a local generation rate $G\left(z,E,\hat{u},\vec{x},t_{x}\right)$. Here, *z* denotes the type of particle, such as *γ*-rays, protons, *α*-particles, heavy ions, etc. With heavy ions further defined by their atomic number and mass number, *E* is the particle energy, $\hat{u}$ is a unit vector specifying the particle direction, $\vec{x}$ is a vector specifying a point of origin on the world surface or within the world volume, and *t_x_* is the time at which the flux is sampled at location $\vec{x}$. The units of the flux are, for example, particles per second, per square centimeter, per MeV, per steradian. The units of the volume generation rate are particles per second, per cubic centimeter, per MeV, per steradian. A typical space environment contains many species, z.

The physical behavior of the system is determined by the probability per unit time $P\left(z,E,\hat{u},\vec{x},t_{x};\xi ,t\right)$ that a quantum of type *z*, which has initial energy *E*, direction $\hat{u}$, and position $\left(\vec{x},t_{x}\right)$, will result in an effect during the interval *dt* at time *t*. The argument *ξ* represents a set of configuration parameters on which this probability may depend. For example, in the RPP model, the size of the RPP and the critical energy *E_c_* are such parameters. Given these three definitions—flux, generation rate, and effect probability—the SER Rate at time *t* for configuration parameters *ξ* can be expressed as:

(1) $$\begin{array}{l} R_{e}\left(\xi ,t\right)=\sum_{z}\int_{All\;E}dE\int d\Omega \oint dS\left(-\hat{n}\left(\vec{x}\right)\cdot \hat{u}\right)\;\times H\left(-\hat{n}\left(\vec{x}\right)\cdot \hat{u}\right)\\ \;\times \int_{-\infty }^{t}dt_{x}\Phi \left(z,E,\hat{u},\vec{x},t_{x}\right)P_{e}\left(z,E,\hat{u},\vec{x},t_{x};\xi ,t\right)\\ \;+\sum_{z}\int_{All\;E}dE\int d\Omega \int dx^{3}\int_{-\infty }^{t}dt_{x}\Phi_{i}\left(z,E,\hat{u},\vec{x},t_{x}\right)P_{e}\left(z,E,\hat{u},\vec{x},t_{x};\xi ,t\right) \end{array}$$

Here, *H*(*x*) is the Heaviside unit step function. Reading from the inside out, the integrals in the first term are over all times *t_x_* prior to *t*; over the whole of the world surface area *S*; over all particle directions Ω, where *d*Ω denotes the differential solid angle in the $\hat{u}$ direction; and over all particle energies *E*. In the second term, the surface integral is replaced by a volume integral over all of the volume enclosed by the world surface. In both terms, the sum is over all species in the respective radiation environment. In the first term, the step function *H*(*x*) constrains the surface and angular integrals to contributions for which $\hat{n}\left(\vec{x}\right)\cdot \hat{u}<0$(that is, inward) [28]. The minus sign appears because the world surface is described by an outward normal vector, while only particles on the world surface that are directed inward are capable of producing an effect. The second term contains no such constraints because spontaneous emission of radiation in any direction within the volume defined by the world surface is potentially significant.

Next, based on the intrinsic characteristics of radiation effects, several forms of Equation (1) are presented under different assumed conditions:

**Assumption** **1.** *The local generation rate of internal radiation sources is zero.*

**Assumption** **2.** *Particle flux is relatively time independent.*

For nearly all scenarios of interest regarding SER, there is no apparent relationship between the duration of SER and variations in average flux. Therefore, it can be assumed that the flux is time independent. Combined with Assumption 1, this allows Equation (1) to be transformed into:

(2) $$\begin{array}{l} R\left(\xi ,t\right)=\sum_{z}\int_{All\;E}dE\int d\Omega \oint dS\left(-\hat{n}\left(\vec{x}\right)\cdot \hat{u}\right)\\ \;\;\;\;\;\;\;\;\;\;\;\;\;\;\;\;\times H\left(-\hat{n}\left(\vec{x}\right)\cdot \hat{u}\right)\Phi \left(z,E,\hat{u},\vec{x}\right)\\ \;\;\;\;\;\;\;\;\;\;\;\;\;\;\;\;\times \int_{-\infty }^{t}dt_{x}P\left(z,E,\hat{u},\vec{x},t_{x};\xi ,t\right) \end{array}$$

**Assumption** **3.** *Radiation transport and effects are instantaneous, and the effect probability P is independent of time.*

When both the total time for radiation transport and the occurrence time of the effects are very short, this type of effect is referred to as an “instantaneous” effect [27]. In the limit case of instantaneous effects, it can be expressed using the Dirac delta function:

(3) $$\begin{array}{l} P\left(z,E,\hat{u},\vec{x},t_{x};\xi ,t\right)=\\ \;\;\;\;\;\;\;\;\;\;\;\;\underset{\varepsilon \to 0}{\lim}P\left(z,E,\hat{u},\vec{x},t_{x};\xi \right)\delta \left(t_{x}-t+\varepsilon \right) \end{array}$$

Thus, Equation (2) can be further simplified to:

(4) $$\begin{array}{l} R\left(\xi ,t\right)=\sum_{z}\int_{All\;E}dE\int d\Omega \oint dS\left(-\hat{n}\left(\vec{x}\right)\cdot \hat{u}\right)\\ \;\;\;\;\;\;\times H\left(-\hat{n}\left(\vec{x}\right)\cdot \hat{u}\right)\Phi \left(z,E,\hat{u},\vec{x}\right)P\left(z,E,\hat{u},\vec{x},t;\xi \right) \end{array}$$

In this case, it is clear that if the effects are independent of time, then the SER Rate is a constant:

(5) $$\begin{array}{l} R\left(\xi \right)=\sum_{z}\int_{All\;E}dE\int d\Omega \oint dS\left(-\hat{n}\left(\vec{x}\right)\cdot \hat{u}\right)\\ \;\;\;\;\;\;\;\;H\left(-\hat{n}\left(\vec{x}\right)\cdot \hat{u}\right)\Phi \left(z,E,\hat{u},\vec{x}\right)P\left(z,E,\hat{u},\vec{x};\xi \right) \end{array}$$

**Assumption** **4.** *The particle radiation environment is homogeneous and isotropic.*

Since the flux is isotropic, the particle flux is no longer a function of the starting point and direction, that is:

(6) $$\Phi \left(z,E,\hat{u},\vec{x}\right)=\Phi \left(z,E\right)$$

The prediction of the error rate for a specific effect is obtained by constructing the appropriate probability distribution function and performing the specified integrals. The starting point for calculating the error rate may be Equations (1), (2), (4), and (5), depending on the specific situation.

### 2.2. The Projected Cross-Sectional Area

In order to establish a correspondence between the aforementioned general SER Rate equation and other equations involving cross-sections, the concept of the sensitive volume familiar in SER studies is extended by introducing the concept of the interaction volume. The interaction volume is the region where physical processes that can directly lead to observable effects occur. It is clear that the sensitive volume is a special form of the interaction volume, but the concept of the interaction volume is more general, especially in the fields of nuclear and particle physics. For example, if the effect is a nuclear reaction, the interaction volume is a sphere with a radius equal to the sum of the radii of the two interacting nuclei. When the interaction volume is described by a cross-section, whether differential or integral, the following assumptions are involved:

**Assumption** **1.** *The effect is instantaneous and time independent.*

**Assumption** **2.** *Interactions only occur within the interaction volume and not before the incident particles reach the interaction volume.*

This assumption implies that the world volume can be reduced to conform to the interaction without losing generality.

**Assumption** **3.** *The trajectories of the incident particles are all parallel and uniformly distributed over the projected area of the interaction volume.*

Thus, the flux distribution associated with a particle beam can be expressed as:

(7) $$\Phi (z,E,\hat{u},\vec{x})=\Phi_{0}\delta (E-E_{0})\delta^{2}(\hat{u}-{\hat{u}}_{0})\delta_{zz_{0}}$$

Here, Φ_0_ is the flux in particles/(cm^2^·s), which is assumed to be independent of $\vec{x}$, in accordance with assumption #3; *δ* and *δ*^2^ are the one- and two-dimensional Dirac delta functions; and the subscripted is the Kronecker delta.

Combined with hypothesis # 1, substitute Equation (7) into Equation (5), and according to the Dirac Delta function equivalence relationship equation and the properties of the Kronecker Delta function [29], Equation (5) can be further simplified:

(8) $$R(\xi )=\Phi_{0}\oint dS(-\hat{n}(\vec{x})\cdot {\hat{u}}_{0})H(-\hat{n}(\vec{x})\cdot {\hat{u}}_{0})\;\times P(z_{0},E_{0},{\hat{u}}_{0},\vec{x};\xi )$$

**Assumption** **4.** *The beam intensity is uniform within the sampled cross-sectional area and is independent of the initial position point.*

That is:

(9) $$P(z_{0},E_{0},{\hat{u}}_{0},\vec{x};\xi )=P(z_{0},E_{0},{\hat{u}}_{0};\xi )$$

Thus, Equation (8) is transformed into:

(10) $$\begin{array}{l} R\left(z_{0},E_{0},{\hat{u}}_{0};\xi \right)=\Phi_{0}P\left(z_{0},E_{0},{\hat{u}}_{0};\xi \right)\\ \;\;\;\;\;\;\;\;\;\;\;\;\;\;\;\;\;\;\;\;\;\;\;\;\;\;\times \oint dS\left(-\hat{n}\left(\vec{x}\right)\cdot {\hat{u}}_{0}\right)H\left(-\hat{n}\left(\vec{x}\right)\cdot {\hat{u}}_{0}\right) \end{array}$$

where the full integral represents the projected area of the interaction volume surface in the ${\hat{u}}_{0}$ direction, denoted as $S_{N}\left({\hat{u}}_{0}\right)$. Therefore:

(11) $$R\left(z_{0},E_{0},{\hat{u}}_{0};\xi \right)=\Phi_{0}P\left(z_{0},E_{0},{\hat{u}}_{0};\xi \right)S_{N}\left({\hat{u}}_{0}\right)$$

According to the definition of the interaction cross-section as the ratio of the effect rate to the flux:

(12) $$\sigma \left(z_{0},E_{0},{\hat{u}}_{0};\xi \right)\equiv \frac{R\left(z_{0},E_{0},{\hat{u}}_{0};\xi \right)}{\Phi_{0}}$$

The correspondence between the effect probability and the projected area can be expressed as follows:

(13) $$P\left(z_{0},E_{0},{\hat{u}}_{0};\xi \right)=\frac{\sigma \left(z_{0},E_{0},{\hat{u}}_{0};\xi \right)}{S_{N}\left({\hat{u}}_{0}\right)}$$

In engineering, $\sigma \left(z_{0},E_{0},{\hat{u}}_{0};\xi \right)$ represents the SER data measured in ground tests, while $P\left(z_{0},E_{0},{\hat{u}}_{0};\xi \right)$ is an expression concerning a set of configuration parameters *ξ* related to SER in integrated circuits. Here, in order to correspond with the cross-section $\sigma \left(z_{0},E_{0},{\hat{u}}_{0};\xi \right)$, $S_{N}\left({\hat{u}}_{0}\right)$ will be referred to as the projected cross-section or intrinsic cross-section.

From the expressions of Equations (12) and (13), we can observe:

(1) For effects that essentially have unit probability $P\left(z_{0},E_{0},{\hat{u}}_{0};\xi \right)≃1$, such as SEUs induced by highly ionizing heavy ions, the size of the SER cross-section is equal to the projected area $S_{N}\left({\hat{u}}_{0}\right)$ of the interaction volume in the ${\hat{u}}_{0}$ direction.
(2) For integrated circuits constructed from basic units with the same SER Rate (or sensitivity), the reliability distribution of the projected cross-section $S_{N}\left({\hat{u}}_{0}\right)$ can be physically characterized using the Weibull function, owing to the reliability of previous data processed using this function. At this point, the exact value of the configuration parameter *ξ* in Equation (17) can be determined through the best Weibull fit of $S_{N}\left({\hat{u}}_{0}\right)$. Of course, for other cases when circuits are specified, the projected cross-section $S_{N}\left({\hat{u}}_{0}\right)$ must also correspond to some exact distribution function, although it does not necessarily have to satisfy Weibull distribution.
(3) During high-energy particle irradiation, the circuit exhibits a certain degree of saturation characteristics, meaning $P\left(z_{0},E_{0},{\hat{u}}_{0};\xi \right)=1$. At this point, the cross-section $\sigma \left(z_{0},E_{0},{\hat{u}}_{0};\xi \right)$ is equal to the projected area $S_{N}\left({\hat{u}}_{0}\right)$ of the interaction volume/world surface in the ${\hat{u}}_{0}$ direction. Of course, $P\left(z_{0},E_{0},{\hat{u}}_{0};\xi \right)$ can also be greater than 1, indicating a supersaturation characteristic, which corresponds to a situation where one incident particle induces multiple effects, such as Multiple Bit Upsets in SEU.
(4) When the particle energy and type are fixed, the size of the projected area $S_{N}\left({\hat{u}}_{0}\right)$ is determined by the configuration parameter set *ξ* related to the effects on the circuit and the initial position parameters ${\hat{u}}_{0}$ of the incident particles. Therefore, if the position of the incident particles is fixed or unchanged, the distribution of $P\left(z_{0},E_{0},{\hat{u}}_{0};\xi \right)$ can be used to infer the consistency of the configuration parameter set *ξ* related to SER, providing important performance evaluation means for radiation-hardened integrated circuit designers.
(5) Equations (12) and (13) provide important bases for evaluating the radiation-hardened performance of circuits in engineering. In practice, the effect cross-section of the circuit can be directly calculated based on test data using the definition of Equation (12), such as the SEU cross-section, single-event transient cross-section, single-event burnout cross-section, etc. Then, according to Equation (13), the projected area $S_{N}\left({\hat{u}}_{0}\right)$ of the interaction volume within the circuit in different ${\hat{u}}_{0}$ directions can be calculated, which represents the overall radiation-hardened performance of the circuit.
(6) The derivation process of Equations (12) and (13) does not require specifying the shape of the interaction volume. The effect rate is only related to the projected area of the interaction volume and the effect probability; thus, this model has good universality.

### 2.3. SER Rate Prediction

When the radiation environment is known, Equation (1) can be used to calculate the occurrence rate of any SER, and its probability $P\left(z,E,\hat{u},\vec{x},t_{x};\xi ,t\right)$ can be determined. It is equally applicable to individual transistors, functional IP modules, entire integrated circuits, or large systems. For engineering purposes, to obtain conservative predictions with high confidence, or at least known confidence levels, the fundamental issue is the appropriate approximation of the physical system (assumption conditions). For space SER, Equation (11) can be directly extended into a very important relationship:

$$R\left(\xi \right)=\sum_{All\;E}\sum_{z}\sum_{\hat{u}}\Phi \left(z,E,\hat{u},\vec{x}\right)P\left(z,E,\hat{u},\vec{x};\xi \right)S_{N}\left(\hat{u}\right)$$

That is, summing is over all particle types *z*, all energy fluxes *E*, and all directions of particles. Here, $\Phi \left(z,E,\hat{u},\vec{x}\right)$ represents the differential directional flux of particles in a specific direction $\hat{u}$, energy *E*, and particle type *z*.

Assuming that the spatial particle radiation environment is uniform and isotropic, this equation can be further simplified in conjunction with Equation (6):

(14) $$R\left(\xi \right)=\sum_{All\;E}\sum_{z}\sum_{\hat{u}}\Phi \left(z,E\right)P\left(z,E,\hat{u};\xi \right)S_{N}\left(\hat{u}\right)$$

This equation outlines the steps for estimating the on-orbit SER Rate using projected cross-sections: First, define the expression $P\left(z,E,\hat{u};\xi \right)$, which contains a set of configuration parameters *ξ* related to the probability of circuit effects. Then, based on ground test data, obtain the distribution function $S_{N}\left(\hat{u}\right)$ that reflects the intrinsic SEU characteristics of the circuit through best fitting. Finally, according to the characterization of space particle properties, such as particle flux Φ(*z*,*E*), the on-orbit effect rate of microelectronic circuits can be estimated using Equation (14).

## 3. Example Applications of Rate Prediction

Here, we choose a spherical coordinate system where the z-axis is aligned with the normal to the circuit plane. The unit vector $\hat{u}$ of the incident particle direction can be characterized by the azimuthal angle *φ* in the (ZOY) plane and the inclination angle *θ* in the (XOY) plane, which are used to constrain the direction of the incident particles. Referring to the relevant literature [30], $P\left(z,E,\hat{u};\xi \right)$ is defined (of course, it can also be another expression) as:

(15) $$\begin{array}{l} P\left(z,E,\hat{u};\xi \right)\equiv P\left(\theta ,\varphi \right)\\ P\left(\theta ,\varphi \right)\;=\sqrt{\left({A_{P}}^{2}\cos^{2}\varphi +B^{2}\sin^{2}\varphi \right)\sin^{2}\theta +\cos^{2}\theta } \end{array}$$

Clearly, the set {*A_P_*, *B*} represents a group of configuration parameters related to the probability of circuit SER mentioned in Equation (14), that is, *ξ* = {*A_P_*, *B*}. The values of *A_P_* and *B* can be determined through a best fitting method.

Equation (14) can be equivalently transformed into a triple integral concerning *E*, *θ*, and *φ*:

(16) $$\begin{array}{l} R\left(\xi \right)=\sum_{z}\int \int \int dE\sin\theta d\varphi d\theta \;\\ \;\;\;\;\;\;\;\;\;\;\;\times \;\Phi \left(z,E\right)P\left(z,E,\hat{u};\xi \right)S_{N}\left(\hat{u}\right) \end{array}$$

If each summation term in the equation represents an isotope from the periodic table, then calculating all the elements in the periodic table would yield hundreds of summation terms.

Since the data set obtained from ground accelerator simulation experiments pertains to LET (Linear Energy Transfer) and SER Rate, in order to directly utilize this data, we will combine the method proposed by Heinrich for calculating LET spectra to equivalently convert the energy E in Equation (14) into another quantity, LET. During this equivalent transformation process, the following assumptions need to be made:

**Assumption** **1.** *The energy deposited in the sensitive volume equals the energy loss of the particles passing through that sensitive volume, and the magnitude of the energy loss can be calculated using its LET.*

For very short particle track lengths, this assumption may not hold [31]. This is due to the finite range of secondary electrons may cause some of the energy lost by ions to escape from the sensitive volume.

**Assumption** **2.** *Ions with the same LET produce the same effects.*

This assumption implies that charged particles can be grouped and processed using the LET spectrum calculation method proposed by Heinrich [32].

The experimental basis for this assumption is limited; in fact, some exceptions have been discovered, such as two ions with different velocities but the same LET and different track structures. Although it currently appears that most exceptions have a negligible impact on SER Rate predictions, some laboratories are investigating this issue.

**Assumption** **3.** *Variations in LET along the ion track within the sensitive volume can be neglected.*

This assumption is primarily used to exclude the following issue: ions with the same LET may have different ranges, thereby depositing different amounts of energy within the same volume. Range effects are a potential problem in accelerator simulations, but they are not encountered in the high-energy environments found in space.

Based on the above assumptions, Equation (16) can be further equivalently transformed into a single triple-integral calculation:

(17) $$\begin{array}{l} R\left(\xi \right)=\int_{0}^{\infty }\int_{0}^{2\pi }\int_{0}^{2\pi }d\varphi d\theta dL\\ \;\;\;\;\;\;\;\;\;\;\;\;\;\times \Phi \left(L,\theta ,\varphi \right)P\left(\theta ,\varphi \right)S_{N}\left(\theta ,\varphi \right)\sin\theta \end{array}$$

Since the flux is isotropic (a typical assumption for calculating heavy ion effect rates), the LET spectrum is the same in all directions. Therefore, the integral over angles in the triple integral of the SER prediction Equation (17) can be moved inward, and:

$$\Phi \left(L,\theta ,\varphi \right)=4\pi \Phi \left(L\right)$$

Here, $\Phi \left(L,\theta ,\varphi \right)$ is referred to as the differential omnidirectional flux.

Furthermore, if we denote:

(18) $$S_{AVG}\left(L\right)=\frac{2}{\pi }\int_{0}^{\frac{\pi }{2}}\int_{0}^{\frac{\pi }{2}}P\left(\theta ,\varphi \right)S_{N}\left(\theta ,\varphi \right)\sin\theta d\varphi d\theta$$

Here, $S_{AVG}\left(L\right)$ *S_AVG_* is referred to as the average directional cross-section related to the effect probability. If the particle flux is symmetric both vertically and horizontally, then the average directional cross-section $S_{AVG}\left(L\right)$ can be further simplified as:

(19) $$S_{AVG}\left(L\right)=\frac{2}{\pi }\int_{0}^{\frac{\pi }{2}}\int_{0}^{\frac{\pi }{2}}P\left(\theta ,\varphi \right)S_{N}\left(\theta ,\varphi \right)\sin\theta d\varphi d\theta$$

Thus, SER prediction Equation (17) transforms to:

(20) $$R\left(\xi \right)=4\pi \int_{0}^{\infty }\Phi \left(L\right)S_{AVG}\left(L\right)dL$$

Thus, based on the effect probability function $P\left(z,E,\hat{u};\xi \right)$, a general SER prediction model has been derived. Next, we will utilize ground heavy ion test data to predict the on-orbit error rate of two types of space-grade integrated circuits and provide a detailed prediction process.

### 3.1. Heavy Ion Experiment

In the next section, we will provide examples of overturn rate prediction for two test devices. The device information is shown in Table 1. First, use the best fit between the cross-section measured from the small-angle test and the directional cross-section obtained from Equation (13) to determine the two fitting parameters *A_P_* and *B* in (15). In order to facilitate the calculation of these two parameters, the following two points should be focused on in the experimental scheme: first, in addition to the directional effect, other factors, such as counting statistical restrictions, particle range, or different test conditions, must result in the discrete distribution of the test data being small enough. The purpose is to clearly distinguish the direction dependence of the sectioning. Second, in the process of measuring the test section, the range of the test ion must be long enough. Within the range of the observed tilt angle, the direction dependence of the section must be clearly observed. The typical practice is to change the tilt angle θ between 0 ° and 60 °, φ taking 0 ° and 90 °, respectively. Then, the Weibull function is selected to best fit the direction section, extrapolate the direction section to a larger angle, and obtain the saturated direction section of the two devices. Finally, combined with the specific space orbit environment, the estimated error rate of the distribution is obtained.

Both heavy ion test campaigns were performed at the SER irradiating Facility on the HI-13 Tandem Accelerator in the China Institute of Atomic Energy, Beijing, and the Heavy Ion Research Facility in Lanzhou (HIRFL) in the Institute of Modern Physics, Chinese Academy of Sciences, which supplied high-energy ions. The devices were irradiated by the C, F, Cl, Ge, and Bi particles listed in Table 2. Provided in the table are the particles, LET, the incident tilt angle θ, the incident azimuth φ, and the SEU cross-sections σ. Applied flux was measured within ±10%, and the beam homogeneity was within ±10%. The typical heavy ion fluence rate was ~1 × 10^4^ ions/cm^2^/s for the SER test. The incident tilt angle was varied from 0° to 60°, and the incident azimuth was taken to be 0° and 90°, respectively. In the table, the unit of LET is MeV·cm^2^/mg, the unit of θ and φ is degree, and the unit of σ is /cm^2^.

### 3.2. Heavy Ions Results

**Figure 1:**
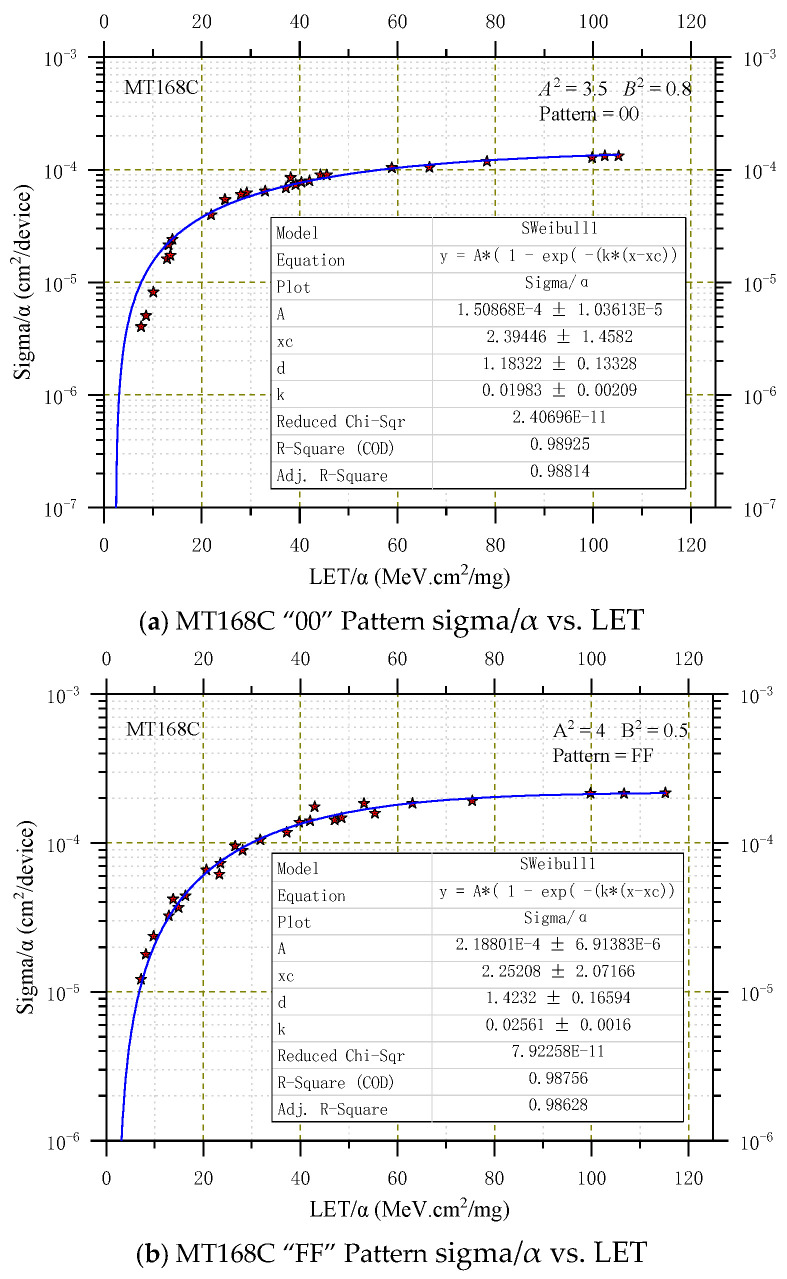
SWeibull1 fit for MT168C heavy ion.

**Figure 2:**
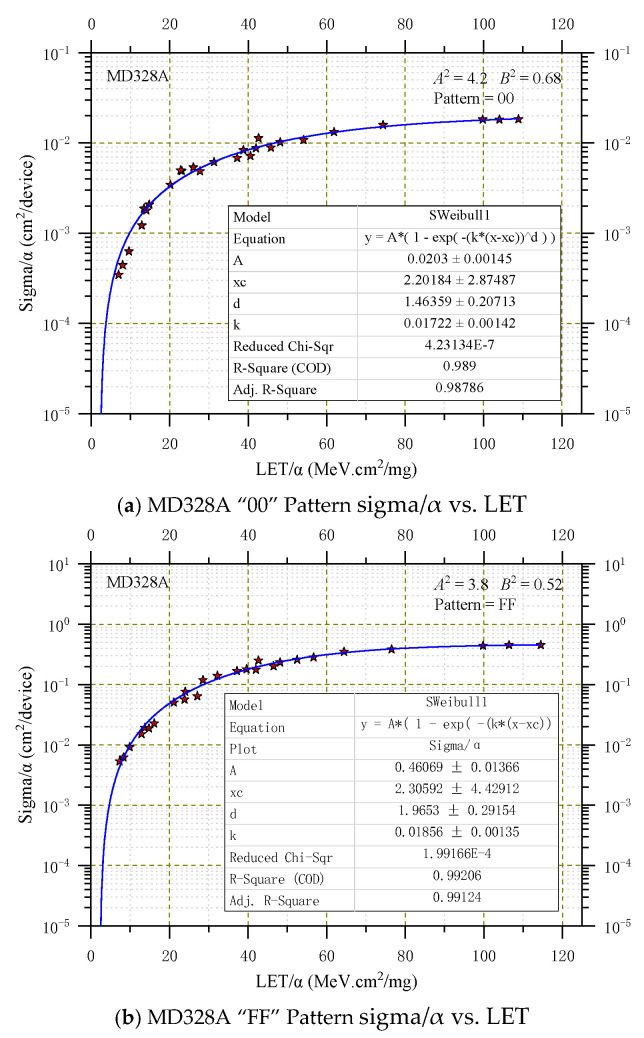
SWeibull1 fit for MD328A heavy ions SEU cross-sections as a function of LET.

A complete specification of the directional cross-section requires not only values for *A_P_* and *B* but also that the normal-incident cross-section be specified. Assuming that Equation (13) is correct, it is convenient to fit points with Weibull curves. The normal-incident cross-section curve is obtained with the least squares fit optimizing Weibull parameters, which were provided in the inset of Figure 1 and Figure 2, where the ordinate sigma/α represents the normal SEU cross-section obtained after the data σ in Table 2 undergoes transformation by Equation (13), and *A_P_*^2^ and *B*^2^ are the corresponding parameters in transformation Equation (15). Note that *A*^2^ is not the square of *A*, as in the Weibull function.

The smooth curves shown in Figure 1 for the normal-incident cross-section of MT168C under two patterns, ‘00’ and ‘FF’, represent the best fit that can be found. But it probably is the simplest and is slightly conservative at the lower LETs, where its curvature does not conform to that indicated by the data points. The corresponding Weibull fitting parameters were automatically labeled on Figure 1a,b during the fitting process in Origin 2022 software. When the internal memory stored the ‘00’ pattern, the Weibull parameters were obtained: A = 1.51 × 10^−4^, X_C_ = 2.40, k = 1.98 × 10^−2^, and d = 1.18 approximately; the parameters obtained from the best fit for α(θ,φ) are A^2^ = 3.5 and B^2^ = 0.8. When the internal memory stored the ‘FF’ pattern, the Weibull parameters were obtained: A = 2.19 × 10^−4^, X_C_ = 2.25, k = 2.56 × 10^−2^, and d = 1.42 approximately; the parameters obtained from the best fit for α(θ,φ) are A^2^ = 4.0 and B^2^ = 0.5.

Figure 2 shows the heavy ion in the normal-incident SEU cross-section of MD328A under two patterns, ‘00’ and ‘FF’. The normal cross-section sigma/α VS LET satisfies the SWeibull1 function very well. Based on the Weibull fitting parameters labeled on Figure 2a,b, when the internal memory stored the ‘00’ pattern, the four Weibull parameters were obtained: A = 2.03 × 10^−2^, X_C_ = 2.20, k = 1.72 × 10^−2^, and d = 1.46 approximately; the parameters for α(θ,φ) are A^2^ = 4.2 and B^2^ = 0.68. When the internal memory stored the ‘FF’ pattern, the Weibull parameters were obtained: A = 4.61 × 10^−1^, X_C_ = 2.31, k = 1.86 × 10^−2^, and d = 1.96 approximately; the parameters for α(θ,φ) are A^2^ = 3.8 and B^2^ = 0.52.

From Figure 1 and Figure 2, it can be observed that the single-event sensitivity is significantly higher when the graph is in the ‘FF’ pattern compared to the ‘00’ pattern. This difference may stem from the slightly greater contribution of n-channel transistors to SEU compared to p-channel transistors [33,34].

### 3.3. SEU Prediction

**Figure 3:**
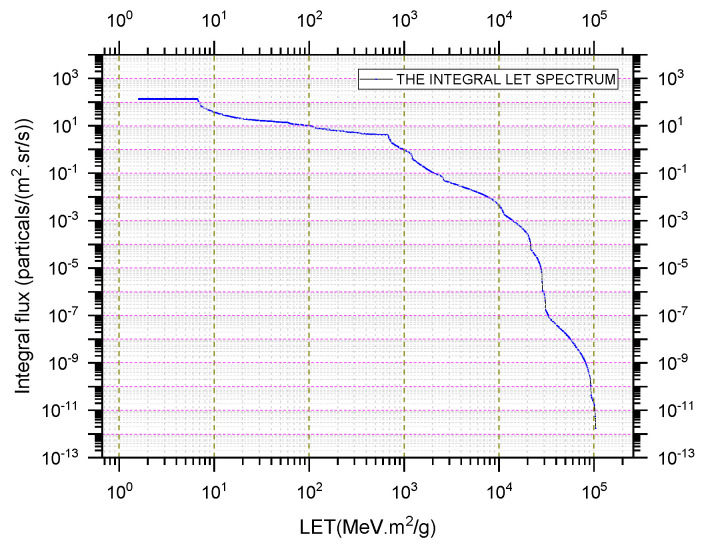
Integral flux spectrum of GCR after passing through 3 mm aluminum.

When predicting the on-orbit SEU rate of the two devices, the interplanetary weather condition considered is galactic cosmic rays (GCRs) only [35], the atomic number of the heaviest element to be included in the LET spectra is 92, and the atomic number of the lightest element to be included in the LET spectra is 2. The integrated flux after passing through 3 mm aluminum is shown in Figure 3. The radiation resistances of these two circuits were evaluated separately. The LET value corresponding to 10% of the saturated cross-section is taken as the LET threshold [36]. When writing MT168C device in modes ‘00’ and ‘FF’, the SEU rate is 6.01 × 10^−6^/device-day and 9.31 × 10^−6^/device-day, respectively. When device MD328A writes graphics ‘00’and ‘FF’, the SEU rate is 1.48 × 10^−4^/device-day and 1.16 × 10^−3^/device-day, respectively.

## 4. Conclusions

In this paper, we have employed foundational knowledge of probability theory, spatial analytic geometry, and vector algebra to establish a SER Rate prediction model. This approach differs slightly from the traditional mathematical foundations of SER prediction models and varies in the number and extent of simplified physical assumptions; some physical assumptions are even not considered at all.

When applying the MC method for numerical simulation prediction, the general model for SER Rate can represent a highly multi-dimensional integral, which is widely regarded as the most effective numerical simulation method in such cases. Furthermore, there is clear evidence that moderately increasing the descriptive details of energy deposition and charge collection can lead to accurate SER Rate predictions, even in complex compositions.

We validated our ideas through two application examples. Based on specific assumptions about the model, known processes, system application characteristics, and analysis of experimental data, we provided a comprehensive thought process, along with corresponding estimation results. This aims to help radiation effects engineers fully understand and apply the aforementioned model.

## Figures and Tables

**Table 1:** Test device information.

Device	Process	Capacity (Kbits)	Unit Structure	Bias (V)
MT168C	28 nm Bulk silicon CMOS process	16 × 8	6 T	0.85
MD328A	28 nm Bulk silicon CMOS process	32 × 8	Double DICE	0.85

**Table 2:** The particles and the SEU cross-section at HI-13 and HIRFL.

Particles	LET	θ	φ	σ_MT168C_		σ_MD328A_	
				00	FF	00	FF
F	4.06	0	0	0.00	0.00	0.00	0.00
Cl	12.9	0	0	1.62 × 10^−5^	3.24 × 10^−5^	1.22 × 10^−3^	1.52 × 10^−2^
Ge	37.2	0	0	6.89 × 10^−5^	1.18 × 10^−4^	6.80 × 10^−3^	1.68 × 10^−1^
Br	42	0	0	7.95 × 10^−5^	1.41 × 10^−4^	8.75 × 10^−3^	1.78 × 10^−1^
Bi	99.8	0	0	1.28 × 10^−4^	2.16 × 10^−4^	1.82 × 10^−2^	4.41 × 10^−1^
F	4.06	30	0	0.00	0.00	0.00	0.00
F	4.06	45	0	0.00	0.00	0.00	0.00
F	4.06	60	0	0.00	0.00	0.00	0.00
Cl	12.9	30	0	1.04 × 10^−5^	3.13 × 10^−5^	8.44 × 10^−4^	1.20 × 10^−2^
Cl	12.9	45	0	7.56 × 10^−6^	2.83 × 10^−5^	7.16 × 10^−4^	9.60 × 10^−3^
Cl	12.9	60	0	6.82 × 10^−6^	2.19 × 10^−5^	6.39 × 10^−4^	9.39 × 10^−3^
Ge	37.2	30	0	7.93 × 10^−5^	1.18 × 10^−4^	6.53 × 10^−3^	1.55 × 10^−1^
Ge	37.2	45	0	8.09 × 10^−5^	1.16 × 10^−4^	7.89 × 10^−3^	1.19 × 10^−1^
Ge	37.2	60	0	6.73 × 10^−5^	1.19 × 10^−4^	6.33 × 10^−3^	8.93 × 10^−2^
Br	42	30	0	8.23 × 10^−5^	1.39 × 10^−4^	8.25 × 10^−3^	1.83 × 10^−1^
Br	42	45	0	9.04 × 10^−5^	1.51 × 10^−4^	8.64 × 10^−3^	9.90 × 10^−2^
Br	42	60	0	9.25 × 10^−5^	1.11 × 10^−4^	9.15 × 10^−3^	9.94 × 10^−2^
Bi	99.8	30	0	1.51 × 10^−4^	2.54 × 10^−4^	2.11 × 10^−2^	5.01 × 10^−1^
Bi	99.8	45	0	1.57 × 10^−4^	2.92 × 10^−4^	2.14 × 10^−2^	5.44 × 10^−1^
Bi	99.8	60	0	1.76 × 10^−4^	2.86 × 10^−4^	1.99 × 10^−2^	4.99 × 10^−1^
F	4.06	30	90	0.00	0.00	0.00	0.00
F	4.06	45	90	0.00	0.00	0.00	0.00
F	4.06	60	90	0.00	0.00	0.00	0.00
Cl	12.9	30	90	2.10 × 10^−5^	3.93 × 10^−5^	1.80 × 10^−3^	1.80 × 10^−2^
Cl	12.9	45	90	1.64 × 10^−5^	3.19 × 10^−5^	1.64 × 10^−3^	1.64 × 10^−2^
Cl	12.9	60	90	2.21 × 10^−5^	3.50 × 10^−5^	1.81 × 10^−3^	1.81 × 10^−2^
Ge	37.2	30	90	8.28 × 10^−5^	1.29 × 10^−4^	7.98 × 10^−3^	1.69 × 10^−1^
Ge	37.2	45	90	6.99 × 10^−5^	1.52 × 10^−4^	6.59 × 10^−3^	2.19 × 10^−1^
Ge	37.2	60	90	7.15 × 10^−5^	1.13 × 10^−4^	9.84 × 10^−3^	1.61 × 10^−1^
Br	42	45	90	8.55 × 10^−5^	1.28 × 10^−4^	8.15 × 10^−3^	2.04 × 10^−1^
Br	42	60	90	8.29 × 10^−5^	1.46 × 10^−4^	8.89 × 10^−3^	2.08 × 10^−1^
Bi	99.8	30	90	1.29 × 10^−4^	2.01 × 10^−4^	1.74 × 10^−2^	4.24 × 10^−1^
Bi	99.8	45	90	1.26 × 10^−4^	1.88 × 10^−4^	1.68 × 10^−2^	3.95 × 10^−1^

SELs were detected in any steps.

## Data Availability

The original contributions presented in the study are included in the article, further inquiries can be directed to the corresponding author.
